# Alkaloid-based regimen is beneficial for acute myeloid leukemia resembling acute promyelocytic leukemia with NUP98/RARG fusion and RUNX1 mutation: A case report

**DOI:** 10.1097/MD.0000000000022488

**Published:** 2020-10-02

**Authors:** Wei Wei, Qiuju Liu, Fei Song, He Cao, Mengmeng Liu, Yan Jiang, Yanchun Li, Sujun Gao

**Affiliations:** a Department of Hematology, Cancer Center, the First Hospital of Jilin University, Changchun; b Peking High Trust Diagnostics, Co., Ltd., Peking, China.

**Keywords:** acute myeloid leukemia, chemotherapy, NUP98/RARG fusion, resembling acute promyelocytic leukemia

## Abstract

**Rationale:**

Some acute myeloid leukemia (AML) patients present with features mimicking the classical hypergranular subtype of acute promyelocytic leukemia (APL) but without the typical promyelocytic leukemia/retinoic acid receptor α (PML/RARα) rearrangement. Herein, we report an AML patient resembling APL but with nucleoporin 98/retinoid acid receptor gamma gene (NUP98/RARG) fusion transcript and Runt-related transcription factor 1 (RUNX1) mutation.

**Patient concerns:**

An 18-year-old male presented at the hospital with a diagnosis of AML.

**Diagnoses:**

The patient was diagnosed with bone marrow examination. Bone marrow smear displayed 90.5% promyelocytes. Fluorescence in situ hybridization analysis failed to detect the PML/RARα fusion transcript or RARα amplification. While real-time polymerase chain reaction showed positivity for the NUP98/RARG fusion transcript. G-banding karyotype analysis showed a normal karyotype.

**Interventions:**

The patient showed resistance to arsenic trioxide and standard 3 + 7 chemotherapy, but eventually achieved complete remission through the Homoharringtonine, Cytarabine, and Aclarubicin chemotherapy.

**Outcomes:**

These measures resulted in a rapid response and disease control.

**Lessons:**

Acute myeloid leukemia with the NUP98/RARG fusion gene and the RUNX1 mutation may be a special subtype of AML and may benefit from the alkaloid-based regimen.

## 1 Introduction

Acute promyelocytic leukemia (APL) is characterized by the presence of the chromosomal translocation t(15;17)(q24;q21) and/or the resulting PML/RARα chimeric protein.^[1]^ However, approximately 1.3% of acute myeloid leukemia (AML) patients present with features mimicking the classical hypergranular subtype of APL but without the typical *PML/RAR*α rearrangement.[^2^,^3^] Among them, a subtype of retinoic acid receptors (*RARs), RARG,* has been reported to be fused with at least 3 alternative partner genes, including *CPSF6, PML,* and *NUP98.* [^4^,^5^,^6^] Herein, we report a nucleoporin 98-retinoic acid receptor gamma (*NUP98/RARG)* gene fusion with a Runt (Runt domain)-related transcription factor 1 (*RUNX1*) mutation in AML mimicking APL; it was sensitive to aalkaloid-based combination but insensitive to arsenic trioxide (ATO) or anthracycline.

## 2 Case Report

**Figure 1:**
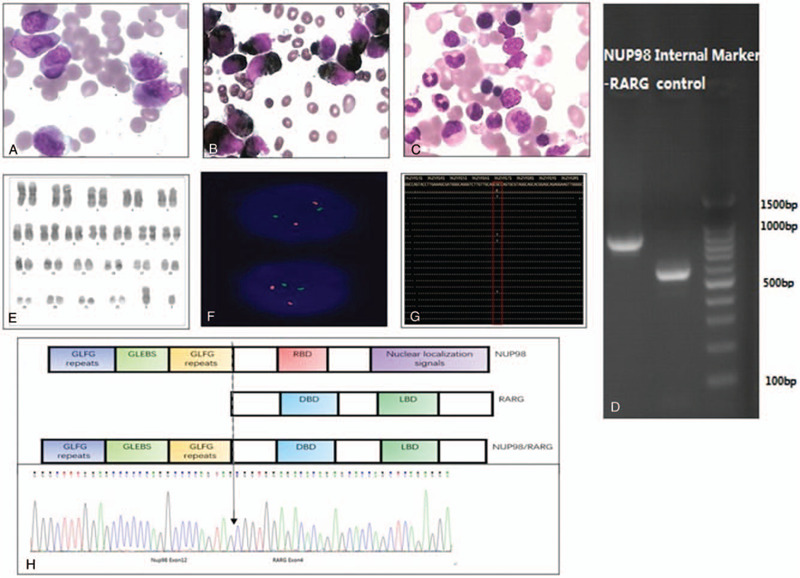
Morphology, karyotyping, FISH, RT-PCR, NGS, and molecular analysis of *NUP98/RARG* fusion. (A) ×400, May Grunwald-Giemsa stain and (B) POX of a bone marrow smear showing promyelocytes with a hypergranulated cytoplasm; several nuclei are invaginated. C, Next-generation sequencing showed the NM_001754(*RUNX1*):c.319C > A (p.R107S) mutation. D, A G-banded karyotype of the aberrant clone showing 46, XY. E, Interphase FISH using the PML/RARα dual-color, dual-fusion translocation probe indicated the absence of the normal PML/RARα. C karyotype. F, Schematic representation of the NUP98/RARG fusion protein. It had preserved the DNA binding domain (DBD) and ligand binding domain (LBD). The arrows indicate the breakpoint and fusion sites of the NUP98/RARG gene. G, Electrophoresis of the RT-PCR products from this patient showed *NUP98-RARG* fusion transcripts. H, Diagram of *NUP98-RARG* fusion gene. FISH = fluorescence in situ hybridization, NGS = next-generation sequencing, RT-PCR = real-time polymerase chain reaction.

An 18-year-old male was admitted to our department with 2-week fatigue and fever. Laboratory workup revealed a leukocyte count of 5310/μL, a hemoglobin level of 10.1 g/dL, and a platelet count of 43,000/μL with 68% atypical promyelocytes. Bone marrow smear displayed 90.5% promyelocytes with morphologic features resembling the classical hypergranular subtype of acute promyelocytic leukemia (Fig. 1A). These cells were strongly positive for peroxidase upon staining (Fig. 1B). The immunophenotype of the blasts was positive for CD117, CD13, CD33, CD9, CD64, CD123, and cMPO but negative for HLA-DR, CD34, CD38, CD11b, and B-cell and T-cell markers. Based on the typical morphology and immunophenotype, the suspected diagnosis was APL, the patient was submitted to intravenous arsenic trioxide (0.15 mg/kg/d) combined with oral all-trans retinoic acid (ATRA)(25 mg/m^2^/d) on the first day of his admission. Five days later, the patient complained of weight gain and headache with an increasing white blood cell count, which indicated he might develop retinoic acid differentiation syndrome, and the ATRA treatment was discontinued. However, after collecting the last genetic results 13 days later, we excluded the diagnosis of typical APL. G-banding karyotype analysis showed negativity for the t(15;17) (q24;q21) translocation but a normal karyotype (Fig. 1D and E). Fluorescence in situ hybridization analysis was performed using a *PML-RARα* dual-color dual-fusion probe (Fig. 1F) according to the manufacturer's protocols, but it failed to detect the *PML/RARα* fusion transcript or *RARα* amplification. Multiple nested reverse transcription polymerase chain reactions (PCRs) were performed to detect 43 fusion transcripts, including *PML/RARα, PLZF/RARα, NUMA1/RARα*, *STAT5b/RARα*, *PAKARIA/RARα, NPM1/RARα,* and *FIPIL1/RARα,* which were negative. Targeted next-generation sequencing of the entire coding sequences of 110 known or putative mutational gene targets in hematologic malignancies identified a 31.69% mutation ratio of the *RUNX1*: c.319C > A(p.R107S) gene (Fig. 1C). Meanwhile, repeated bone marrow smears and flow cytometry (FCM) analyses still showed the existence of 86% abnormal promyelocytes.

**Figure 2:**
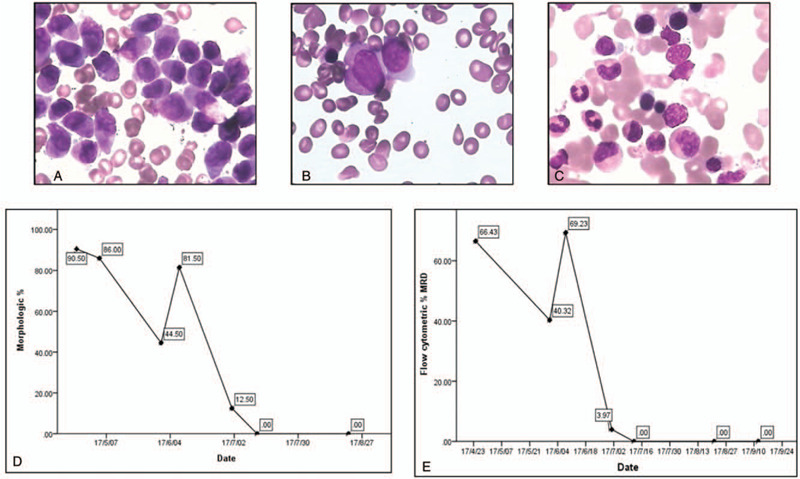
CR and MRD after HAA regimen chemotherapy. A–C, Morphology change before chemotherapy and 13 days and 24 days after the chemotherapy. D, E, MRD change during treatment. After CR was induced by HAA, the patient had stayed complete remission and negative for MRD for more than 3 months. CR = complete reemission, HAA = Homoharringtonine, Cytarabine and Aclarubicin.

Therefore, we stopped the use of arsenic trioxide and switched to a standard 3 + 7 chemotherapy schedule (60 mg/m^2^ doxorubicin, d1-3; 100 mg/m^2^ cytarabine, d1-7 continuously). During this course, he showed fibrinolysis with a mild low serum fibrinogen level. The evaluation of bone marrow morphology showed there were still 44.5% and 81.5% abnormal promyelocytes (Fig. 2A) respectively, on the 14th day and the 21st day after the finish of the chemotherapy. Then, we changed the chemotherapy regimen to Homoharringtonine, Cytarabine and Aclarubicin (HAA) (2 mg/m^2^ homoharringtonine, d1-7; 14 mg/m^2^ aclarubicin, d1-7, combined with 100 mg/m^2^ cytarabine, d1-7 continuously). Meanwhile, another real-time polymerase chain reaction (RT-PCR) showed positivity for the *NUP98/RARG* fusion transcript (Fig. 1G). The *NUP98/RARG* mRNA was reverse transcribed into cDNA using random primers, and PCR was performed using the following primers: forward: 5’-GGG CTT GGT GCA GGA TTT GG-3’, and reverse: 5’-TGG GTC CGG TTC AGG GTC AGC-3’ (NUP98: NCBI reference sequence: NM_016320.4; RARG: NCBI reference sequence: NM_001042728.2). These primers were also used to amplify the fusion transcript breakpoints. On the 14th day after the finish of chemotherapy with HAA, morphology showed 12.5% abnormal promyelocytes with differentiation signs (Fig. 2B), and FCM showed 3.97% abnormal myeloid blasts positive for CD117, CD33, CD34, and HLA-DR. At last, both morphology (Fig. 2C) and FCM were negative and CBC had recovered 1 week later. He achieved complete remission with a decreased level of the *NUP98/RARG* fusion gene (0.1%). Then, the patient received another cycle of HAA followed by 1 cycle of high-dose cytarabine (2 g/m^2^, q12 h, d1, d3, and d5) as consolidation therapy. During this period, the patient maintained complete reemission (CR) with morphology (Fig. 2D) and MRD negativity with flow cytometry (Fig. 2E). However, because of personal problems, the patient refused to receive any further treatment. Three months later he relapsed with the same morphologic, immunophenotypic, and molecular features displayed at diagnosis but with 8 new point mutations in WT1. He still refused to receive any therapy for his leukemia. Then, he developed an anal abscess with coagulopathy. Ten days later, he died of a severe infection.

The patient provided written informed consent for the publication of these case details, and the consent procedure was approved by the ethic committee of the first hospital of Jilin University.

## 3 Discussion

This was the third human AML case harboring the *NUP98/RARG* rearrangement to date. It was supposed that the *NUP98* 5-region encoding the glycine–leucine–phenylalanine–glycine-repeat and the GLE2p-binding Sequence-like motifs were fused to the 3-region of *RARG*, which included the DNA- and ligand-binding domains of the gene (Fig. 1H). Similar to other *RARG* fusion gene with AML, this kind of acute leukemia showed the clinical feature with coagulopathy, and the morphology, immunophenotyping were mimicking with APL. But the treatment result was totally different. In this case, our patient showed resistance to the anthracycline-containing regimen, different from the first patient who reached CR with a standard 7 + 3 chemotherapy approach.^[6]^ And the sensitivity to ATRA was controversial. The first patient relapsed with the same features and the researchers did in vitro studies on the relapsed *NUP98/RARG* fusion and reported that it confers resistance to ATRA treatment.^[7]^ However, another in vivo experiment in murine models showed that cells transformed by the *NUP98/RARG* fusion were extremely sensitive to ATRA treatment.[^8^,^9^] In our case, similar to the more recently reported case with the *PML/RARG* and *NUP98/RARG* fusion gene, the sensitivity to ATRA treatment was not established due to the early discontinuation of ATRA therapy.^[5]^ Among the 7 patients who were reported with the *RARG* rearrangement,[^3^,^10^,^11^] none showed clear sensitivity to ATRA. But we can confirm the resistance to ATO in this *NUP98/RARG* fusion gene-positive AML patient, similar to other reports.[^3^,^4^]

*RUNX1* mutations occur in 13.7% of normal chromosome karyotype AML patients but rarely in APL patients.[^1^,^3^] In AML patients, the *RUNX1* mutation is correlated with poor clinical outcomes, even when treatment with intensive therapeutic strategies is performed.^[12]^ Our patient showed resistance to the standard 3 + 7 induction chemotherapy but benefitted from a homoharringtonine (HHT)-based combination. HHT is a natural alkaloid isolated from various Cephalotaxus species. It can bind to and increase the level of myosin-9 in myeloid leukemia to induce the apoptosis of leukemia cells.^[13]^ A recent study showed that HHT treatment alone caused potent inhibition of AML cell growth/survival in vitro and substantial suppression of AML progression in vivo, and such inhibitory effects are likely attributed to HHT-induced cell cycle blockage and apoptosis, as well as enhanced myeloid differentiation.^[14]^ AHHT-based combination regimen was shown to be highly effective in some subtypes (FLT3 and t(8;21)) of AML patients.[^15^,^16^] Our patient had maintained CR for a half year but relapsed due to early treatment discontinuation.

How to treat AML patients with *NUP98/RARG* remains uncertain, but it is challenging because of coagulation abnormality and fatal bleeding risk, which demand a strong supply of blood products to avoid early death. The patient in this report experienced coagulopathy after undergoing chemotherapy with daunorubicin and cytarabine (DA), which suggests some similar characteristics with typical APL. However, the detailed biological function of *NUP98/RARG* needs to be investigated in the future. In summary, we reported 1 case of acute myeloid leukemia with the *NUP98/RARG* fusion gene and the *RUNX1* mutation that resembled acute promyelocytic leukemia in regards to its morphologic and immunologic features. Herein, we confirm that this is a new subtype of acute myeloid leukemia that is insensitive to ATO and anthracycline, but can benefit from the alkaloid-based regimen.

## Acknowledgments

This work was supported by the Science and Technology Department of Jilin province, Project No 20180101132JC.

## Author contributions

Wei Wei, Qiuju Liu, and Sujun Gao contributed to the design of the article. Wei Wei, Qiuju Liu, Fei Song, He Cao, and Mengmeng Liu contributed to the analysis of data and wrote the manuscript. Yan Jiang, and Yanchun Li contributed to the collection of data. Sujun Gao was the research advisor.
